# Patients with Achilles Tendon Rupture Are Prone to Develop Ventricular Arrhythmia

**DOI:** 10.3390/jcm12103583

**Published:** 2023-05-21

**Authors:** Volkan Gür, Furkan Yapici, Uğur Küçük, İzzet Özay Subaşi, Mehmet Burak Gökgöz, Reşit Karaköse, Nizamettin Koçkara

**Affiliations:** 1 Department of Orthopedics and Traumatology, Faculty of Medicine, Erzincan University, 24180 Erzincan, Turkey; 2 Department of Cardiology, Faculty of Medicine, 18 Mart University, 17020 Çanakkale, Turkey

**Keywords:** Achilles tendon rupture, arrhythmia, electrocardiogram, repair, surgery, ventricular repolarization

## Abstract

Background and Objectives: This study aimed to examine the ventricular repolarization (VR) disturbances of patients operated on for acute spontaneous Achilles tendon ruptures (ATRs), by comparing them with a healthy individual control group. Materials and Methods: Between June 2014 and July 2020, a total of 29 patients (28 males, 1 female; mean age: 40 ± 9.78 years; range, 21–66 years) who presented to the emergency department within the first three weeks of injury, and were diagnosed with acute spontaneous ATRs and treated with an open Krackow suture technique, were retrospectively analyzed. Fifty-two healthy individuals (47 males, 5 females; mean age: 39 ± 11.45 years; range, 21–66 years) were recruited as a control group from the cardiology outpatient clinic. Clinical data (demographic features and laboratory parameters (serum glucose, creatinine, hemoglobin, white blood cell count, and lipid profile)) and electrocardiograms (ECGs) were collected from medical records. ECGs were evaluated for heart rate and VR parameters of QRS width, QTc interval, cQTd interval, Tp-e interval, and Tp-e/QT ratio. The clinical data and these ECG parameters were compared between groups. Results: There was no statistically significant difference between groups, regarding clinical data (all *p* < 0.05). Among ECG parameters, heart rate, QRS width, QTc interval, and cQTd interval were similar between groups (all *p* < 0.05). There were two important statistically significant findings of this research: The mean Tp-e interval was longer (ATR group: 72.4 ± 24.7, control group: 58.8 ± 14.5, *p*: 0.01), and the Tp-e/QT ratio was higher (ATR group: 0.2 ± 0.1, control group: 0.16 ± 0.4, *p*: 0.027) in the ATR group. Conclusions: According to the ventricular repolarization disturbances found in this study, patients with ATR may be at a higher risk of ventricular arrhythmia than healthy people. As a result, ATR patients should be assessed for ventricular arrhythmia risk by an expert cardiologist.

## 1. Introduction

The Achilles tendon is the body’s most robust and biggest tendon, but it also gets ruptured the most. The Achilles tendon often ruptures between three and six centimeters above the point where it attaches to the calcaneus [1]. The exact cause of acute spontaneous Achilles tendon ruptures (ATRs) is unknown because most of the patients who sustain a rupture had no prior symptoms such as tenderness, stiffness, discomfort, or any diagnosed disease in the region of the Achilles tendon before the event [2]. The diagnosis of an acute Achilles tendon rupture is typically determined based on the patient’s description of a pop or snap in the posterior aspect of the ankle, as well as the sudden onset of pain. An ankle that is swollen and excruciating, with posterior ecchymosis, is revealed by a physical examination. The inability to rise onto the toes or a heel resistance test, performed by grasping the heel and resisting plantar flexion, demonstrating a lack of plantar flexion force, is indicative of an Achilles tendon rupture. The Thompson test has been proven to be a reliable indicator of a ruptured Achilles tendon. During this test, the calf muscle is compressed with the patient prone and their feet hanging off the end of the examination table. The foot can plantarflex if the tendon is in good condition. The foot can not move significantly, if at all, after being hit. This is indicative of an Achilles tendon rupture [3].

Numerous studies have been conducted on Achilles tendon rupture, but its causes and treatments remain controversial. Although conservative treatments appear to have a higher re-rupture incidence, some authors prefer them. Other treatment options are open primary repair or minimally invasive repair. Minimally invasive surgery is associated with fewer complications and has been shown to be more respectful to the biology of tendon healing, so it is preferred by the majority of experts today [4].

ATR is considered to have a multifactorial etiology [5]. While inflammatory mediators are known to contribute to the initiation and progression of tendon disease in the shoulder, the relative importance and role of inflammation are highly debated in energy-storing tendons such as the Achilles where, historically, the disease was frequently described as “degenerative” [6].

Effectively preventing cardiovascular diseases and reducing cardiac events can be achieved through timely diagnosis. Electrocardiogram (ECG), as a noninvasive diagnostic instrument, remains a clinically simple, easy, and rapid method, and its ST-T ischemic change has significant value in the diagnosis of coronary heart disease (CHD). Over 75% of angina patients have been evaluated using an initial ECG [7].

Increased sympathetic and inflammatory activity directly relates to cardiac arrhythmias and sudden cardiac death, through electrical disturbances during ventricular repolarization. Ventricular repolarization can be evaluated by calculating QT interval, QT dispersion, and T wave changes in electrocardiography (ECG). Especially in recent studies, it has been shown that the Tp-e interval (the interval from the peak to the end of the t wave in ECG) is an indicator of impaired ventricular depolarization, and an increased Tp-e value is a predictor of ventricular arrhythmia [8].

Achilles tendon pathologies are associated with familial hypercholesterolemia and coronary artery disease [9]. Xanthoma of the Achilles tendon is an established risk factor for coronary artery disease (CAD). The thickness of the Achilles tendon (ATT) has been identified as a risk factor for cardiovascular disease. In CAD patients, the presence of ATT has been previously linked to disease severity and plaque susceptibility. Using ATT for risk categorization of subsequent cardiovascular events could prove beneficial [10]. The Achilles tendon collects lipids in people with familial hypercholesterolemia, and thickening of the Achilles tendon causes the tendon to weaken and lose its resistance to stress [11]. Arrhythmias are a significant clinical concern and a common reason for hospitalization in the developing population. Due to their eventful clinical history, and potential cardiovascular issues, they require careful attention [12]. In analyzing the ECGs of ATR patients, we noticed that these patients might also be in cardiac danger in the form of ventricular arrhythmias, in addition to the previously stated Achilles tendon diseases.

Blood lipid levels are modifiable risk factors for atherosclerosis and coronary heart disease, with cholesterol, cholesterol esters, triglycerides, and phospholipids being the major classes of lipoproteins. Excess fatty acids (FA) in the liver are converted into triacylglycerols, which are then packaged into very-low-density lipoprotein (VLDL) and apoproteins. Lipoprotein lipase (LPL) is used to hydrolyze triglyceride contents into FA and VLDL remnants, while LDL, having the apo-B100 apoprotein component, is the major cholesterol carrier in the peripheral circulation. All three types of lipoplasma are major CHD risk factors [13].

To the best of our knowledge, there have been no studies conducted to evaluate the electrophysiologic alterations, particularly ventricular arrhythmias, that occur in patients who have sustained an ATR. The purpose of this study is to determine whether or not patients who have ATRs are at an elevated risk of ventricular arrhythmias associated with the Tp-e interval and the Tp-e to QT ratio. We hypothesize that patients with ATRs are prone to develop ventricular arrhythmia.

## 2. Materials and Methods

This study was performed under the approval of our institution’s ethical review board (Document number: E-21142744-804.99-96216) and was conducted under the Declaration of Helsinki. Informed consent was obtained from each participant before the operations.

In our cross-sectional study, between June 2014 and July 2020, 29 patients (Male/Female: 28/1) operated on for Achilles tendon rupture, and 52 patients (Male/Female: 47/5) without Achilles tendon pathology were included. ATR was diagnosed with a physical examination (Thompson test and gap sign) and ankle MRI imaging. Patients without Achilles tendon pathology, patients with ankle MRI due to ankle pain, and patients with no Achilles tendon pathology were included.

Demographic information of the patients was recorded. Pre-operative ECGs of the patients operated on for acute spontaneous ATR were evaluated. Patients without Achilles tendon pathology were recruited, and cardiac ECGs were performed for these patients (Table 1).

ECGs of all patients, in both groups, were in sinus rhythm. Those with a history of drug use, such as beta-blockers, calcium channel blockers, amiodarone, digoxin, and tricyclic antidepressants that could cause changes in repolarization measurements on ECG were not included in the study (n = 2).

The study did not include those with hyperthyroidism, systolic heart failure, moderate or severe heart valve disease, permanent pacemakers, chronic kidney disease, and electrolyte imbalance (n = 3). Patients with findings on ECGs, such as left and right bundle branch block, pathological q wave, and atrioventricular conduction block on ECGs, were not included in the study (n = 2). ECGs of the patients who did not have sufficient image quality for analysis were not included in the study (n = 2).

We accessed the national health database of our country for each patient and checked whether they had a history of a heart problems and a prior lipid profile blood test. We discovered that none had a reported heart condition history, and all had a blood lipid profile test acquired by each patient’s family physician for routine annual blood parameters control before this study. Despite none of the patients having a reported heart condition history in the national database, this outcome was not objective enough to determine whether they really had a heart problem. To resolve this issue, patients were called for a follow-up visit, for stress ECG, to decide whether or not they had myocardial ischemia. Patients were to undergo stress echocardiography if they had a positive sign of ischemia on the stress ECG. However, none had a positive sign of ischemia on the stress ECG, such as an ST elevation/depression.

One cardiologist (UK) performed the ECG evaluation. An ECG recording protocol was applied by taking 12-lead ECG recordings with a paper speed of 25 mm per second, 10 mm/mV height, and a filter interval of 0.16–100 Hz. All ECGs were scanned and transferred to a personal computer to reduce error measurements and then used for 400% magnification with Adobe Photoshop software. All measurements were carried out on the screen by manual method.

**Figure 1:**
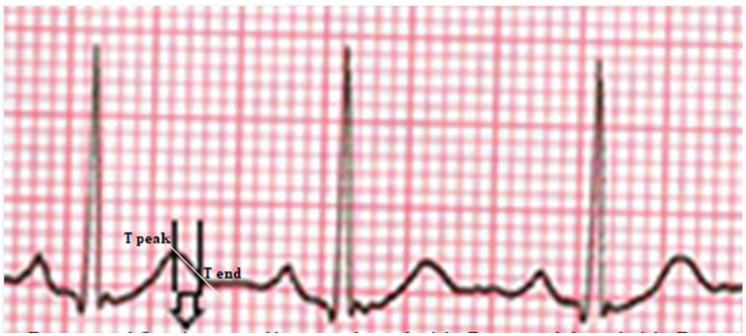
The Tp-e interval was defined as the distance between the peak of the T-wave and the endpoint.

The QT interval was defined as the distance from the beginning of the Q wave to the end of the T wave. The heart rate corrected QT interval (QTc) was calculated using Bazett’s formula [14]. The Tp-e interval was defined as the distance between the peak of the T wave and the endpoint (Figure 1). Tp-e interval measurements were made from precordial leads. The Tp-e interval is a noninvasive index of arrhythmogenesis that is used to measure the dispersion of ventricular repolarization in ECG. It is applied to different cardiac conditions associated with a high risk of sudden cardiac death [15]. QTd was defined as the difference between the minimum and maximum QT interval. cQTd was defined as the difference between the minimum and maximum cQT range. The Tp-e/QT ratio was obtained by proportioning the Tp-e values to the QT values.

### Statistical Analysis

Continuous variables obtained from the analysis were demonstrated as mean ± standard deviation; categorical variables were expressed as percentages and numbers.

All statistical analyzes were performed using SPSS 19.0 (SPSS Inc., Chicago, IL, USA). The Kolmogorov–Smirnov test was used to evaluate the distribution of continuous variables. Independent samples t-test and Mann–Whitney U-test were used to compare continuous variables. The Chi-square test or Fischer exact test was used to compare nominal data. A *p*-value of <0.05 was considered statistically significant. An independent statistician conducted the statistical analyses.

## 3. Results

Our study population comprised 29 patients with Achilles tendon rupture, and 52 healthy control patients. The case and control groups were matched for age, gender, hypertension, diabetes mellitus, smoking, serum glucose value, creatinine, hemoglobin, white blood cells, and lipid profile. There was no statistically significant difference among these parameters between groups (all *p* < 0.05) (Table 1).

Heart rate, QRS width, cQTd, and QTc intervals were not significantly different, compared with respective controls (all *p* < 0.05). Tp-e interval and Tp-e/QT ratio were prolonged in the ATR group, compared to the control group, and this difference was statistically significant (*p* = 0.01, *p* = 0.03, respectively) (Table 2).

## 4. Discussion

The most important finding of this study was that the Tp-e interval and Tp-e/QT ratios were significantly prolonged in the ATR group, compared to the healthy control group, indicating that ATR patients are susceptible to ventricular arrhythmia. Thus, our hypothesis can be accepted.

A rupture in the Achilles tendon can be caused by various factors, including patient-specific circumstances such as lower extremity malalignment, drug use, or inappropriate shoe selection. Research has shown that tendinosis-like features were observed in 77% of patients with Achilles tendon rupture, which is consistent with other medical conditions. Tendinosis can be caused by a range of factors, such as circulatory disorders or injuries from reperfusion. A histological study is necessary to identify these factors. Even though Achilles tendon rupture is considered an acute event, histological research has shown that, even in the setting of an acute rupture, degenerative alterations indicative of Achilles tendinosis are typically detected inside torn tendons. This is the case even though Achilles tendon rupture is considered an acute process. As a result, it is believed that Achilles tendinosis is a factor related to Achilles tendon rupture [16].

Pain in the Achilles tendon should prompt a search for treatable metabolic disorders. Indeed, some authors advocate not only the measurement of plasma cholesterol in all patients presenting with a painful Achilles tendon, but also offer criticism of standard orthopedic textbooks for failing to mention familial hypercholesterolemia as a cause of Achilles tendon pain. Familial hypercholesterolemia is a prevalent autosomal dominant disease with a prevalence of 1 in 500, but approximately three-quarters of cases are not diagnosed until middle age. Heterozygous familial hypercholesterolemia entails a high risk of premature coronary heart disease (>50% risk in men by age 50 and >30% risk in women by age 60) and mortality if undiagnosed and untreated. Conversely, patients whose diagnosis is recognized and who receive intensive early treatment, including lifestyle counseling and statin therapy, can attain a normal life expectancy. Detecting definite heterozygous familial hypercholesterolemia prior to the advent of clinically manifest coronary heart disease is, therefore, of utmost importance [17].

Sudden cardiac death refers to an unexpected and sudden loss of heart function, typically caused by an underlying heart condition. It can occur in people of all ages, but it is most common in adults over the age of 35. Some of the underlying heart conditions that can lead to sudden cardiac death include coronary artery disease, arrhythmias (irregular heart rhythms), and structural abnormalities of the heart [18]. Ventricular arrhythmias are the most common cause of sudden cardiac death because of impaired ventricular repolarization, and numerous factors such as heart failure, coronary artery disease, and electrolyte disorders are the culprits in the etiology [19]. Parameters such as increased QT interval, Tp-e interval, and Tp-e/QT ratio are important indicators showing the deterioration in ventricular repolarization. TpTe/QT values were significantly higher in patients with life-threatening arrhythmias than those without major arrhythmic occurrences. This was independently linked to heart rate and maximal ST elevation, which are associated with the prognosis and severity of myocardial infarction [20].

The mechanism that can cause arrhythmia is complex. Heart failure and arrhythmic genetic diseases (such as Bragada syndrome, long QT syndromes, and hypertrophic cardiomyopathy) may cause changes in ventricular repolarization and arrhythmias triggered by the underlying systemic inflammation [21]. Additionally, fear and sudden unexpected news can cause activation of the sympathetic nervous system and cause fatal ventricular arrhythmias. Similarly, Reich et al. showed that psychological stress could facilitate ventricular arrhythmias [22]. The study involved 62 patients with implanted cardioverter-defibrillators (ICDs) who were monitored for 24 h while they went about their daily activities. The researchers found that there was a higher incidence of ventricular arrhythmias during periods of psychological stress compared to periods of rest.

Studies have demonstrated that systemic disorders have a role in the development of Achilles tendinopathy, as well as ruptures of the Achilles tendon. The connection between hypercholesterolemia and Achilles tendon rupture has not been conclusively established, despite the fact that hypercholesterolemia is a risk factor for Achilles tendinopathy [23]. Cholesterol is an important component of cell membranes and plays a crucial role in many biological processes. However, high levels of cholesterol in the blood (hypercholesterolemia) have been associated with an increased risk of various health conditions, including cardiovascular disease and metabolic disorders. In recent years, researchers have also investigated the relationship between hypercholesterolemia and musculoskeletal disorders, including tendinopathy [24].

Achilles tendon diseases are linked to familial hypercholesterolemia and coronary artery disease [25]. Xanthomas of the Achilles tendon are a well-known indicator of a significant risk of CAD. In addition, increased ATT has been recognized as a cardiovascular disease risk factor. In individuals with CAD, an increase in ATT has been previously associated with disease severity and plaque formation. ATT may be helpful for the risk classification of subsequent cardiovascular events [26]. The softness of the Achilles tendon (AT) can be utilized as an additional sign of lipid accumulation in individuals with familial hypercholesterolemia (FH). In addition to assisting in diagnosing FH, AT softness is associated with the incidence of atherosclerotic cardiovascular disease and the severity of carotid atherosclerosis [27]. AT strain ratio, determined with strain elastography ultrasound, is also a helpful parameter for predicting CAD [28]. In short, a relationship has been observed between coronary artery diseases that cause severe mortality and morbidity, and Achilles tendon pathologies.

Hypercholesterolemia is a CAD risk factor, with 56% of total heart diseases being due to it. It was found that patients had significantly higher total cholesterol levels, and the lipid profile was more disturbed in those with other metabolic conditions. The increased risk of CAD was linked to insulin resistance and central obesity [29]. The management of hypercholesterolemia should include not only the control of cholesterol levels but also the management of other metabolic conditions that may contribute to the development of heart disease. This may involve lifestyle modifications, such as a healthy diet and regular exercise, as well as medications, such as statins, that are used to lower cholesterol levels and reduce the risk of heart disease [30].

In combination with a lipid profile, an ECG can help diagnose coronary ischemia and guide the management of the condition. Abnormalities in the lipid profile, such as hypertriglyceridemia and low levels of HDL, have been linked to many diseases, including obesity, diabetes, and cardiovascular diseases. It has been estimated that the risk of CAD decreases by 2 to 3% for every 1 mg/dL increase in HDL. Elevated levels of triglycerides, fasting and nonfasting, are also independent risk factors for CHD. Low HDL and elevated triglycerides have the highest rate of major coronary events, while serum cholesterol has been shown to be an established CHD risk factor [31,32]. ECG is a diagnostic test that can help identify the presence of coronary ischemia. An ECG measures the heart’s electrical activity and can detect any abnormalities in the heart’s rhythm or function. During an episode of coronary ischemia, the heart muscle may not receive enough oxygen and nutrients, which can cause changes in the heart’s electrical activity that can be detected by an ECG [33]. These changes may include ST-segment elevation/depression, T wave inversion, or the development of Q waves, depending on the severity and duration of the ischemia [34,35]. It is important to note that a normal ECG does not rule out the presence of coronary ischemia, and further testing (stress ECG, stress echocardiogram, and myocardial perfusion scintigraphy) may be necessary if there is a strong suspicion of the condition based on symptoms, risk factors, or other diagnostic tests.

As seen in the studies mentioned above, although there are various studies conducted with the Achilles tendon, the effects of Achilles tendon pathologies on myocardial conduction and their relationship with arrhythmic events have yet to be investigated in studies conducted so far. Similarly, the relationship between Achilles tendon rupture and cardiac involvement has yet to be studied. Hence, this is the first study reporting this relationship.

Our study observed a significant difference in ventricular repolarization parameters in patients with ATR compared to healthy individuals, indicating that physiological ventricular repolarization rhythm may deteriorate in patients with ATR.

In addition to the literature, ATR is a new clinical picture that should be questioned in the etiology of ventricular arrhythmia.

The major strength of this study is that it is the first of its kind to assess ventricular repolarization, among individuals with ATR, in the existing literature. Some significant drawbacks of our study are the relatively small number of patients who underwent ATR surgery, and the uneven distribution of male and female participants. Furthermore, there was no histopathological analysis conducted to determine if the ruptures were caused by degeneration, reduced blood supply, or lipid buildup in the affected Achilles tendons. Extensive prospective studies are needed to determine the predictive value of prolonged Tp-e interval and increased Tp-e/QT ratio in ATR patients for screening ventricular arrhythmia. Future prospective research should find the investigation of biomechanical and psychological factors’ role in the development of ATR to be of interest.

## 5. Conclusions

According to the findings of this study, which showed that people with ATR had disturbances (prolonged Tp-e interval and increased Tp-e/QT ratio) in their ventricular repolarization rhythm, there may be a higher risk of ventricular arrhythmia for these patients. Thus, ATR patients should be assessed by an expert cardiologist for propensity to ventricular arrhythmia.

## Figures and Tables

**Table 1:** Demographic and laboratory features of the patients.

Variables	ATR Group (n = 29)	Control Group (n = 52)	*p*
Age (Years)	40 ± 9.78	39 ± 11.45	0.964
Gender (Male/Female)	28/1	47/5	0.412
HT (Number)	2	8	0.318
DM (Number)	3	8	0.738
Current smoker (Number)	9	10	0.751
Serum glucose (mg/dL)	99.2 ± 19.7	102.2 ± 30.6	0.600
Creatinine (mg/dL)	1 ± 0.3	1 ± 0.3	0.571
Hemoglobin (g/dL)	15.3 ± 1.2	15.9 ± 1.5	0.068
WBC count (×10^3^/μL)	9 ± 3.4	10.2 ± 2.4	0.059
LDL cholesterol (mg/dL)	121.3 ± 49.73	118.19 ± 35.28	0.454
HDL cholesterol (mg/dL)	42.81 ± 11.59	37.91 ± 12.80	0.252
Triglyceride (mg/dL)	174.81 ± 90.51	146.79 ± 74.59	0.368
Total cholesterol (mg/dL)	198.72 ± 67.68	178.76 ± 62.35	0.399

ATR: Achilles tendon rupture, DM: diabetes mellitus, HT: hypertension, WBC: white blood cell, LDL: low-density lipoprotein, HDL: high-density lipoprotein.

**Table 2:** Electrocardiographic characteristics of the study population.

Variables	ATR Group (n = 29)	Control Group (n = 52)	*p*
Heart rate (bpm)	75.7 ± 14	68.9 ± 12.4	0.062
QRS width (ms)	88.7 ± 14.1	92.7 ± 13.9	0.215
QTc interval (ms)	359.1 ± 31.3	351.5 ± 34.5	0.559
cQTd interval (ms)	398 ± 40.2	376.1 ± 59.5	0.074
Tp-e interval (ms)	72.4 ± 24.7	58.8 ± 14.5	0.010
Tp-e/QT ratio	0.2 ± 0.1	0.16 ± 0.4	0.027

Data are given as mean ± SD. bpm: beats per minute, Tp-e: transmural dispersion of repolarization, ms: milliseconds.

## Data Availability

Research data are unavailable due to the privacy and ethical restrictions of our institution.
